# 1p and/or 19q polysomy is an adverse prognostic factor in oligodendrogliomas, and easy to detect by automated FISH

**DOI:** 10.1371/journal.pone.0322809

**Published:** 2025-05-02

**Authors:** Karine Michaud, Peter Vincent Gould, Myreille D’Astous, Claudie Paquet, Stephan Saikali

**Affiliations:** 1 Department of Neurosurgery, Centre Hospitalier Universitaire de Québec, Québec, Canada; 2 Department of Pathology and Molecular Genetics, Centre Hospitalier Universitaire de Québec, Québec, Canada; The University of Texas MD Anderson Cancer Center, UNITED STATES OF AMERICA

## Abstract

**Objective:**

To study the feasibility of automated analysis by FISH technique in the determination of the 1p and/or 19q polysomy in oligodendrogliomas (OGs) and to explore its prognostic value.

**Methods:**

We analyzed a retrospective monocentric series of 145 consecutive OGs with IDH mutation and 1p/19q codeletion. For all cases, automated FISH analyses were performed to determine 1p and/or 19q polysomy status and results were compared to manual analysis to verify the concordance of the two methods. Polysomic status was then compared to clinical and histological data, the CDKN2A deletion status when available, event free survival (EFS) and overall survival (OS).

**Results:**

Our study comprised 79 grade 2 OGs (O2) and 66 grade 3 OGs (O3). Polysomy of 1p and/or 19q was observed in 58 cases (40% of whole cohort) with a significant enrichment in the high grade cohort (59% versus 24%; p < 0,0001) and recurrent cases (55%). A majority of polysomic cases were copolysomic for 1p and 19q (75% of the polysomic cohort) rather than 1p or 19q single polysomy (21% and 4% respectively). Polysomy was correlated to high grade histological criteria of high mitotic and Mib1 proliferative indices (p = 0,002 and p = 0,0005 respectively) and to vascular proliferation (p = 0,0003). Univariate and multivariate analysis showed a significant correlation betwen polysomy and a shorter EFS and OS (p = 0,02 and p = 0,016 respectively). Concordance between manual and automated analysis was almost perfect for both 1p and 19q analysis (96 and 98% respectively, κ = 0,92 and 0,95 respectively). Automated analysis revealed that the large majority of polysomic signatures are represented by a small number of R/G signals (mainly 7 signatures) allowing a very easy implementation to pre-existent FISH platforms analysis software.

**Conclusion:**

1p and/ or 19q polysomy status represent a prognostic factor in OGs and can be easily determined by automated analysis. Our study supports the clinical interest to determine the polysomic status in all primitive or recurrent OGs and underline the benefits of automated analysis which offers a better archive storage and facilitates multicentric comparison.

## Introduction

Since the last two editions of the WHO`s classification of central nervous system, oligodendroglioma (OG) is a very well defined entity and classified as an adult-type diffuse glioma with IDH1 or IDH2 missense mutation combined with whole-arm deletions of 1p and 19q [1]. The allelic loss of 1p and 19q in this glioma corresponds to a balanced translocation involving chromosomes 1 and 19 at their centromeres with maintenance of the der(1;19)(q10; p10) and subsequent loss of the derivative chromosome der(1;19)(p10;q10) [2,3]. The 1p and 19q codeletion is associated with sensitivity to chemotherapy, an increased benefit of adjuvant chemotherapy given after radiotherapy as well as more favorable outcome [4,5]. The main histological features which define high grade OGs (O3) with poor prognosis are the presence of microvascular proliferation and tumoral necrosis with or without palisading [6].

Despite the numerous genomic studies published over the last decade on gliomas in general and on OG in particular, very few new genetic alterations of notable diagnostic or prognostic value in OGs have been identified to date. The most notable of these alterations is undoubtedly the presence of homozygous deletion involving the CDKN2A and/or CDKN2B gene on the 9p. This deletion appears strongly associated with short survival in OGs and could serve as a molecular marker of grade 3 OGs [7]. Genomic analysis of large cohorts of gliomas have also highlighted the prognostic value of PIK3CA mutation [8–10], TCF12 mutation [8,11] and MYC pathway deregulation (by gene locus gain or gene promoter hypomethylation or by downregulation of MYC silencers [12] which appear to be linked to unfavorable outcome in O3.

These recent large scale multicentric studies nevertheless ignored a genetic particularity described in retrospective studies on oligodendroglial tumors using the FISH technique. Indeed, since the beginning of the determination of the 1p and the 19q status in oligodendroglial tumors at early 2000’s and since the strict characterization of OGs by the presence of IDH mutation and 1p/19q codeletion (WHO 2016), FISH represents one of the most widespread techniques to study this codeletion [13]. Retrospective studies of oligodendroglial tumors using FISH have shown the presence of occasional 1q and/or 19p polysomy concurrent with 1p/19q codeletion in a small proportion of tumors [14–18]. This 1p/19q codeletion with polysomy appeared associated with adverse outcome (earlier recurrence and shorter survival) [14,15,18–20] and was considered sufficiently relevant to be mentioned in the last edition of the WHO`s classification of central nervous system [1] with the mention of the need for further studies to be accepted as a relevant prognostic marker.

For more than 20 years, our institution has developed an expertise in the study of 1p and 19q status by FISH technique on paraffin embedded tissue and signal analysis is routinely automatized with a high concordance compared to manual [21]. All our cases undergo a double analysis (one manual done by a trained operator and one automated provided by our platform’s software: i.e., Metafer 4 from Metasystem) and all the images used for this double analysis are stored and archived in our platform hard disk. Given the significant number of OGs in our FISH virtual images archives, we wanted to analyze the presence and the proportion of 1p and/or 19q polysomy within our codeleted cases and see if this polysomy offers additional prognostic value. We also wanted to compare the manual analysis to the automated analysis in the determination of polysomy and to study their level of concordance. Finally in our large and well annotated single-institution cohort we studied the correlation between this polysomy and the clinical data, histological data and available CDKN2A deletion status.

## Materials and methods

### Ethics statement

The Research Ethics Committee of the Centre Hospitalier Universitaire de Québec was consulted for this study (project 2024–7349): S1 Table. Tumor samples were collected and anonymized by the Pathology department of the Centre Hospitalier Universitaire de Québec (Hôpital de l’Enfant-Jésus, Quebec City, Canada).

### Patients and tissue specimens

Formalin fixed and paraffin-embedded (FFPE) tissue from 145 available consecutive cerebral OGs (19 biopsies and 126 surgical resections) diagnosed in our institution between 27/02/1998 and 05/12/2022 were selected for this study. All cases were analyzed by two neuropathologists (PVG and SS) and reclassified according to the recent guidelines of the World Health Organization for central nervous system tumours [1] into 79 O2 and 66 O3. Authors had access to information that could identify individual participants during and after data collection.

### Immunohistochemistry

Immunohistochemistry (IHC) was performed on 4 µm thick FFPE sections with the EnVision^TM^ FLEX+ detection system on Dako Autostainer 48 (Dako, Mississauga, Ontario). Reaction was visualized by EnVision^TM^ FLEX DAB+ Chromogen (Dako, Glostrup, Danemark).

All cases were screened for monoclonal antibodies IDH1 R132H (clone H09; 1/50; Optistain), ATRX (clone D-5; 1/50; Santa Cruz), p53 (clone DO-7; no dilution; Dako) and Ki67 (clone Mib1; no dilution; Dako) with an incubation of 30 min for each.

IDH132H expression was considered as positive (mutated) if at least one positive cell was observed otherwise it was considered negative (wild type). ATRX expression was scored as positive (wild type) if ≥ 10% of cells were positive [22] or negative (mutated). p53 and Ki67 expression was scored as percentage by counting the immunostained nuclei of 100 cells in the most positive area at high power field (HPF = x400; i.e., 0,16 mm^2^). p53 staining was considered positive when ≥50% of tumor cells presented a strong nuclear staining. Ki67 (Mib1) proliferative index was classified into 2 subgroups according to the median of the whole cohort (≤17% and > 17%).

### FISH technique

FISH analysis for 1p, 19q and 9p (CDKN2A) status was performed using commercial probes: Vysis LSI 1p36/1q25, Vysis LSI 19q13/19p13 and LSI CDKN2A (9p21)/CEP 9 (9p11-q11) Dual-Color Probe kits (Abbott Molecular Inc., Abbott Park, Illinois, USA).Targeted chromosomal arm were labelled by a red fluorophore (namely 1p36.32, 19q13.33 and 9p21.3). The opposite chromosomal arm (for 1p and 19q) or centromere (for 9p) serving respectively as control and labelled in green.

5-µm-thick formalin-fixed, paraffin-embedded sections were used for each probe according to our routine FISH procedure as detailed previously [21,23].

Signal acquisition was performed for each slide over the 12 more representative areas. These areas were automatically captured at x400 using a Metasystem station (Zeiss MetaSystems, Thornwood, NY) equipped with a Zeiss Axioplan fluorescent microscope. These acquired images were then used as the basis for manual and automated counting assays. Automated analysis was performed using the Metafer 4 software (Metasystem).

### FISH interpretation

FISH manual analysis was performed by a single observer (SS) on 100 non-overlapping nuclei for green ‘G’ (control) and red ‘R’ (target) signals. Automated analysis was performed on all tumor cells identified by the Metafer 4 software using the algorithm previously established for the analysis of 1p and 19q status [21]. This sampled a mean of 1003 cells (min: 265 – max: 2285 – median: 1011) for chromosome 1, 989 cells (min: 295 – max: 2302 – median: 943) for chromosome 19 and 1122 cells (min: 214 – max: 2437 – median: 1077) for chromosome 9 respectively.

**Fig 1:**
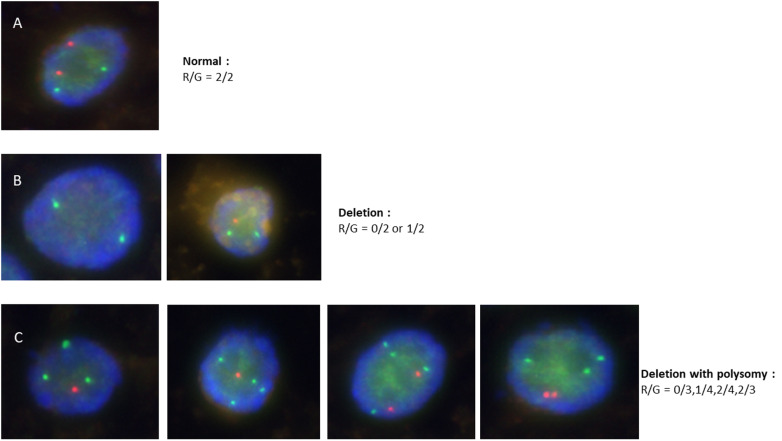
Representative R/G signals (Red spots/Green spots) for normal status **(A)**, deletion status without polysomy (B) and deletion status with polysomy (G ≥ 3) for 1p/1q and 19q/19p **(C)**.

For both 1p and 19q, the cut off were previously set at 55% of total analyzed tumoral cells [21] with an R/G ratio <0.75. The tumor was considered to have a deletion with polysomy if ≥ 30% of nuclei showed three or more signals for 1q and/or 19p [14]: R/G = 0/3, 0/4, 0/5, 2/3, 2/4, 2/5, 3/5, 3/6, 4/8, etc…for example (Fig 1).

For 9p, the cut-off for the deletion status was calculated on a series of 10 non-neoplastic brain tissue samples (from epilepsy surgery cases). This cut-off was calculated using mean +3 SD and was set at 40% for hemizygous deletion status and 30% for homozygous deletion status. Hemizygous deletion is defined as cell with at least two centromeric signals (G ≥ 2) and at least one 9p signal (R ≥ 1): R/G = 1/2, 1/3, 1/4, 2/4, etc….Homozygous deletion is defined as cell with at least one centromeric signal (G ≥ 1) and no 9p signals (R = 0): R/G = 0/1,0/2,0/3,0/4, etc…[24].

### Statistical analyses

Data analysis was performed using GraphPad Prism 10.2.2 software. Chi-square test was performed for group comparisons between clinical (age at diagnosis, sex and recurrence), histological (grade, microvascular proliferation, mitotic index, calcification and Ki67 labelling index) and molecular status data (CDKN2A deletion and 1p and/or 19q deletion with polysomy). Concordance between manual analysis and automated analysis was estimated by calculating Cohen’s kappa coefficient (κ): κ value between 0.6 and 0.8 reflecting a good agreement and κ value >0.8 constituting a high concordance. In order to identify histological and/or molecular data related to event free survival (EFS) and overall survival (OS), survival curves were plotted according to the method of Kaplan and Meier, and the log-rank test was used to assess the statistical significance of differences observed in survival between distinct groups. EFS was defined as the time from diagnosis to the first recurrence. OS was defined as the time from diagnosis to death or last follow-up set until 01/03/2024. For the identification of parameters with an independent prognostic impact on patient overall survival, the Cox regression was used. In the multivariate analysis only those variables that showed a significant impact in the univariate analysis were included. Threshold for statistical significance was set at p < 0.05.

## Results

### Clinical data

Clinical data are summarized in Table 1. Our series include 145 patients with 86 males (59%) and 59 females (41%) with a mean age of 49 years (median = 49 years). Majority of patients underwent open surgery with gross total or partial tumor resection (n = 126; 87%) and few of them got stereotactic biopsies (n = 19; 13%).

**Table 1:** Clinical, histological and molecular data according to the tumor grade and the polysomic status.

		O2	O3	χ2 (p)		No polysomy	Polysomy	χ2 (p)
	Total	(79)	(66)			(87)	(58)	
	(%)	54%	46%			60%	40%	
**Histological grade**					O2	60 (69%)	19 (33%)	**<0,0001**
					O3	27 (31%)	39 (67%)	
**Sex**	Male	50 (63%)	36 (54%)	NS		52 (60%)	34 (59%)	NS
	Female	29 (37%)	30 (46%)			35 (40%)	24 (41%)	
**Age**	≤ 49y	42 (53%)	31 (47%)	NS		49 (56%)	24 (41%)	NS
**(years)**	>49y	37 (47%)	35 (53%)			38 (44%)	34 (59%)	
**Site**	Frontal	60 (76%)	51 (77%)	NS		69 (79%)	42 (73%)	NS
	Parietal	7 (9%)	5 (7%)			6 (7%)	6 (10%)	
	Temporal	8 (10%)	8 (12%)			9 (11%)	7 (12%)	
	Occipital	0	1 (2%)			0	1 (2%)	
	Cingular	4 (5%)	1 (2%)			3 (3%)	2 (3%)	
**Status**	Dead	17 (22%)	41 (62%)	**<0,0001**		28 (32%)	30 (52%)	**0,02**
	Alive	62 (78%)	25 (38%)			59 (68%)	28 (48%)	
**Extent of surgery**	Biopsy	16 (20%)	3 (5%)	**0,005**		12 (14%)	7 (12%)	NS
	Surgery	63 (80%)	63 (95%)			75 (86%)	51 (88%)	
**Recurrence**	No	73 (92%)	43 (65%)	**<0,0001**		74 (85%)	42 (72%)	NS
	Yes	6 (8%)	23 (35%)			13 (15%)	16 (28%)	
**Treatment**	None	36 (46%)	11 (16%)	**0,0004**		32 (37%)	15 (26%)	NS
	Chemotherapy	29 (37%)	33 (50%)			38 (44%)	24 (42%)	
	Radiotherapy	9 (11%)	7 (11%)			10 (11%)	6 (10%)	
	Chemo + Radio	5 (6%)	15 (23%)			7 (8%)	13 (22%)	
**Mitosis**	≤ 5 per 10 HPF	76 (96%)	34 (52%)	**<0,0001**		74 (85%)	36 (62%)	**0,002**
	> 5 per 10 HPF	3 (4%)	32 (48%)			13 (15%)	22 (38%)	
**Vascular**	Endocrinoid	79 (100%)	5 (8%)	**<0,0001**		61 (70%)	23 (40%)	**0,0003**
**proliferation**	Glomeruloid	0	61 (92%)			26 (30%)	35 (60%)	
**Necrosis**	No	79 (100%)	36 (55%)	**<0,0001**		74 (85%)	41 (71%)	NS (0,058)
	Yes	0	30 (45%)			13 (15%)	17 (29%)	
**Calcification**	No	50 (63%)	29 (44%)	**0,03**		53 (61%)	26 (45%)	NS
	Yes	29 (37%)	37 (56%)			34 (39%)	32 (55%)	
**Mib**	≤ 17%	70 (89%)	18 (27%)	**<0,0001**		63 (72%)	25 (43%)	**0,0005**
	> 17%	9 (11%)	48 (73%)			24 (28%)	33 (57%)	
**CDKN2A deletion**	No	58/60 (97%)	42/59 (71%)	**0,0002**		59/67 (88%)	41/52 (79%)	**0,04**
	Homozygot	0	8 (14%)			1 (2%)	7 (13%)	
	Hemizygot	2 (3%)	9 (15%)			7 (10%)	4 (8%)	
**1p/19q polysomy**	No	60 (76%)	27 (41%)	**<0,0001**		87		
**status**	Yes	19 (24%)	39 (59%)				58	
**1p/19q polysomy**	No	60 (76%)	27 (41%)	**<0,0001**		87		
**subgroups**	1p and 19q	15 (19%)	29 (44%)				44	
	1p alone	3 (4%)	9 (14%)				12	
	19q alone	1 (1%)	1 (1%)				2	

Statistically significant: p-values <0.05, NS: not significant, HPF: high power-field, O2: grade 2 oligodendroglioma, O3: grade 3 oligodendroglioma.

For purposes of statistical analysis the tumor location was considered to be the lobe of the brain within which the largest volume of the glioma resided. In our series the majority of cases was located in the frontal lobe (n = 111; 77%) followed by the temporal lobe (n = 16; 11%), the parietal lobe (n = 12; 8%), the cingular lobe (n = 5; 3%) and the occipital lobe (n = 1; 1%). Twenty nine (29) cases among the 145 cases were recurrent tumors (20%).

47 cases had no post-operative treatment (32%), 16 cases received post-operative radiotherapy only (11%), 62 cases received chemotherapy only with PCV or Temozolomide (43%) and 20 were treated with adjuvant PCV or Temozolomide radiochemotherapy (14%).

The cohort EFS mean was of 57 months (min = 1; max = 286 and median = 41) whereas the cohort OS mean was of 87 months (min = 1; max = 297 and median = 73). Eighty seven (87) patients were still alive (60%) and 58 were dead (40%).

### Histological data

In our series, according to the WHO 2021 CNS5 classification, 79 (54%) patients were classified into O2 and 66 (46%) into O3: Table 1. All cases presented an IDH mutation (137 IDH1 mutation and 8 IDH2 mutation), a preservation of ATRX expression (mean expression > 80% of tumoral cells) and no hyper-expression of p53 (mean expression always weak and < 5% of tumoral cells) on immunohistochemistry analysis. As defined by the WHO, glomeruloid microvascular proliferation (MVP) and necrosis were absent in O2 and present only in O3 population (p < 0.0001 for both). High mitotic index (>5 per 10 HPF) and high MIB1 proliferative index (>17%) were linked to the O3 subgroup (p < 0.0001 for both respectively). Tumoral calcifications were also more present in the O3 subgroup (p = 0.03).

### Molecular data

CDKN2A deletion status was available for 119 patients (60 O2 and 59 O3): Table 1. For the remaining 26 patients, technical artifacts and lack of tissue available did not allow reliable interpretation. Deletion of the CDKN2A gene was found in 19 patients (16% of all cohort) with 8 homozygotes (7% of the cohort) and 11 hemizygotes (9% of the cohort) for the gene. According to the WHO 2021 CNS5 classification, all the homozygote patients were classified into O3 subgroup (8/8 = 100%) and the majority of the hemizygote patients also belonged to this subgroup (9/11 = 82%). The correlation of the CDKN2A deletion with the histological grade appeared very strong in univariate analysis (p = 0.0002).

In our cohort, 1p/19q deletion with polysomy was present in 19 O2 (24%) and 39 O3 (59%) with a significant statistical predominance in the high grade subgroup (p < 0,0001). The majority of these cases presented a copolysomy for chromosomes (Chr) 1 and 19 (44/58 = 76%). The others had mainly a single Chr1 polysomy (12/58 = 21%) rather than a single Chr19 polysomy (2/58 = 3%). The correlation of polysomy with the high grade remains significant despite the stratification into these 3 subgroups (p < 0,0001).

In univariate analysis, polysomy of Chr1 and/or Chr19 appeared strongly correlated to high mitotic and MIB1 proliferative index (p = 0,002 and p = 0,0005 respectively) and also to the presence of vascular proliferation (p = 0,0003): Table 1. We observed a trend to correlation with tumoral necrosis (p = 0,058). The frequency of polysomy was higher in recurrent cases (16/29 = 55%) than in primary cases (42/116 = 36%) with a p = 0.03. On the other hand, there was no significant difference of recurrence between polysomic and non-polysomic subgroups (28% versus 15%, p = non-significant). The large majority of recurrent case with polysomy presented a copolysomy (15/16 = 94%) and only one case presented a single 1p polysomy. For 13 of our recurrent cases, virtual images archives were also available for primitive tumor and allowed a visual comparison of the polysomic status evolution. Five(5) of these cases presented no polysomy at initial and recurrent tumor (38%), three (3) cases presented copolysomy at initial and recurrent tumor (23%), four (4) cases presented a copolysomy at the recurrence only (31%) and one case (1) presented a single 1p polysomy at the initial glioma and no polysomy detected at the recurrence (8%).

Polysomy was correlated to the presence of CDKN2A deletion (homozygote or hemizygote status): p = 0,04. There was no significant correlation between polysomy and gender, age, anatomical site, presence of calcification or the type of treatment (Table 1).

### Correlation of clinical, histological and molecular data with EFS

**Fig 2:**
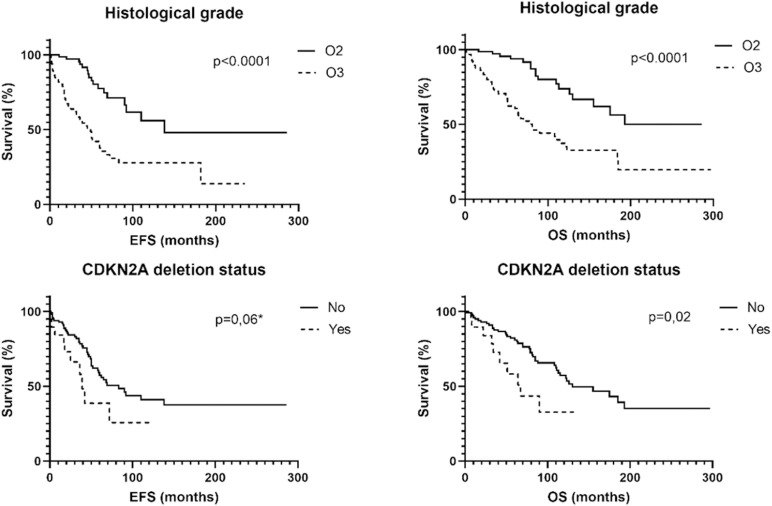
Event Free Survival (EFS) and Overall Survival (OS) according to the histological grade and to the chromosomal status. O3 anaplastic oligodendrogliomas are associated with shorter EFS and OS (raw 1). The presence of a CDKN2A gene deletion on 9p (both homozygous and heterozygous deletion) is associated with a shorter OS and a trend to a shorter EFS (raw 2).

Kaplan-Meier survival curve analysis revealed a 1-year EFS likelihood of 99%, 5-years survival likelihood of 78%, 10-years survival likelihood of 56%, 15 years survival likelihood of 48% and 20 years survival likelihood of 48% for the O2 cohort. For O3 cohort, EFS analysis revealed a 1-year survival likelihood of 82%, 5-years survival likelihood of 38%, 10-years survival likelihood of 27%, 15 years survival likelihood of 27% and 20 years survival likelihood of 14% (Fig 2, p < 0,0001).

**Fig 3:**
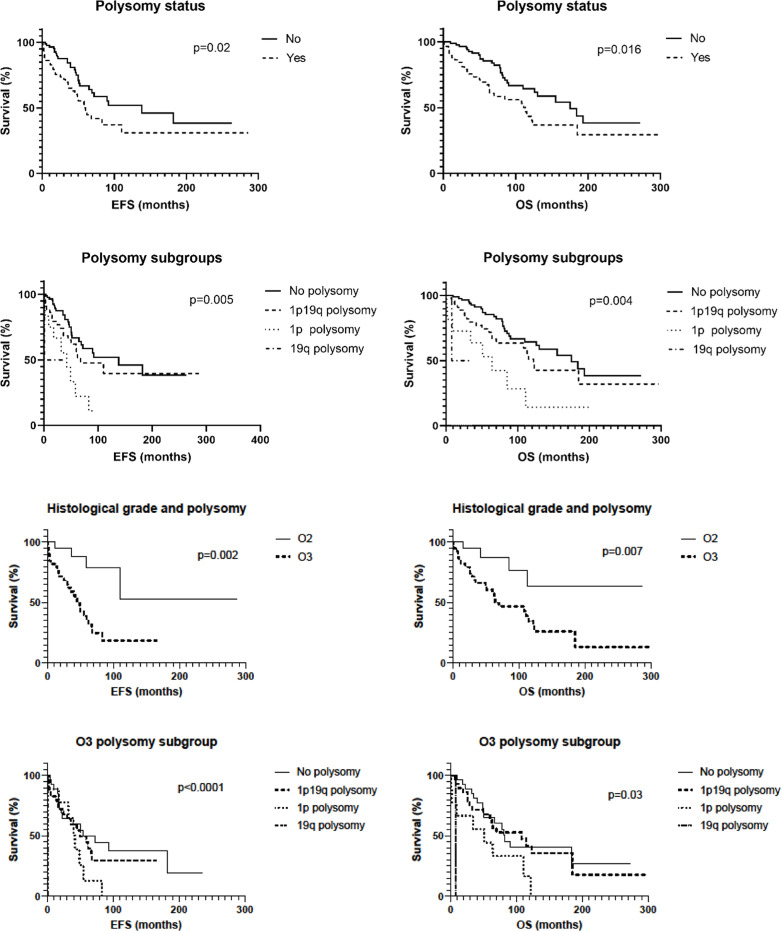
Event Free Survival (EFS) and Overall Survival (OS) according to the 1p and/ or 19q polysomic status. Polysomy is associated to a shorter EFS and OS (raw 1) regardless to 1p/19q copolsomy or single 1p or 19q polysomy status (raw 2). Polysomy is strongly associated to histological high grade (raw 3) and within the O3 cohort, presence of polysomy still corelated to shorter EFS and OS (raw 4).

Recurrent OGs appeared to correlate with a shorter EFS (110 months versus 30, p < 0,0001): Table 2, Fig 3. There was no correlation between older age, anatomical site, the type of treatment and the presence of calcification with EFS in our series (Table 2, Fig 3).

**Table 2:** Correlation between clinical, histological and molecular data with survival in univariate analysis.

		EFS		OS	
		Median survival	p	Median survival	p
		(months)		(months)	
**Histological grade**	O2	138	**<0,0001**	undefined	**<0,0001**
	O3	49		81	
**Age**	≤ 49y	90	NS	185	NS (0,056)
**(years)**	>49y	83		122	
**Site**	Frontal	83	NS	130	NS
	Parietal	59		undefined	
	Temporal	46		111	
	Occipital	36		63	
	Cingular	undefined		undefined	
**Recurrence**	No	110	**<0,0001**	175	**<0,0001**
	Yes	30		50	
**Treatment**	Non	93	NS	155	NS
	Chemotherapy	110		123	
	Radiotherapy	58		85	
	Chemo + Radio	62		122	
**Mitosis**	≤ 5 per 10 HPF	138	**<0,0001**	184	**<0,0001**
	> 5 per 10 HPF	42		55	
**Vascular**	Endocrinoid	138	**<0,0001**	193	**<0,0001**
**proliferation**	Glomeruloid	49		81	
**Necrosis**	No	110	**<0,0003**	184	**<0,0001**
	Yes	42		60	
**Calcification**	No	90	NS	130	NS
	Yes	62		155	
**Mib**	≤ 17%	138	**<0,0002**	184	**<0,0001**
	> 17%	50		70	
**CDKN2A deletion**	No	83	NS (0,06)	130	**0,024**
	Yes	39		67	
**CDKN2A subgroups**	Normal	83	NS	130	**0,049**
	Homozygot	25		undefined	
	Hemizygot	39		51	
**1p/19q polysomy**	No	138	**0,02**	175	**0,016**
**status**	Yes	60		113	
**1p/19q polysomy**	No	138	**0,005**	175	**0,004**
**subgroups**	1p and 19q	68		122	
	1p alone	42		64	
	19q alone	19		22	
**O3 and polysomy**	No	54	NS	82	NS
**status**	Yes	45		64	
**O3 and polysomy**	No	54	**<0,0001**	82	**0,03**
**subgroups**	1p and 19q	50		108	
	1p alone	42		51	
	19q alone	1		8	
**Polysomy and grade**	O2	undefined	**0,002**	undefined	**0,007**
	O3	45		64	

Statistically significant: p-values <0.05, NS: not significant, HPF: high power-field, O2: grade 2 oligodendroglioma, O3: grade 3 oligodendroglioma, EFS: Event free survival, OS: Overall survival.

On univariate analysis, high mitotic and proliferative indices (p < 0,0001 and p < 0,0002 respectively), vascular proliferation (p < 0,0001) and tumor necrosis (p < 0,0003) were correlated to a shorter EFS: Table 2 and Fig 2.

CDKN2A deletion presented a trend toward a shorter EFS (median of 83 months versus 39, p = 0,06). Patients who had no CDKN2A deletion had a median EFS of 93% at 1-year, 57% at 5-years, 41% at 10 years, 38% at 15 years and 38% at 20 years whereas patients who had CDKN2A deletion (homozygous or hemizygous) had a median EFS of 84% at 1-year, 39% at 5-years and 26% at 10 years (p = 0,06): Table 2 and Fig 2.

**Fig 4:**
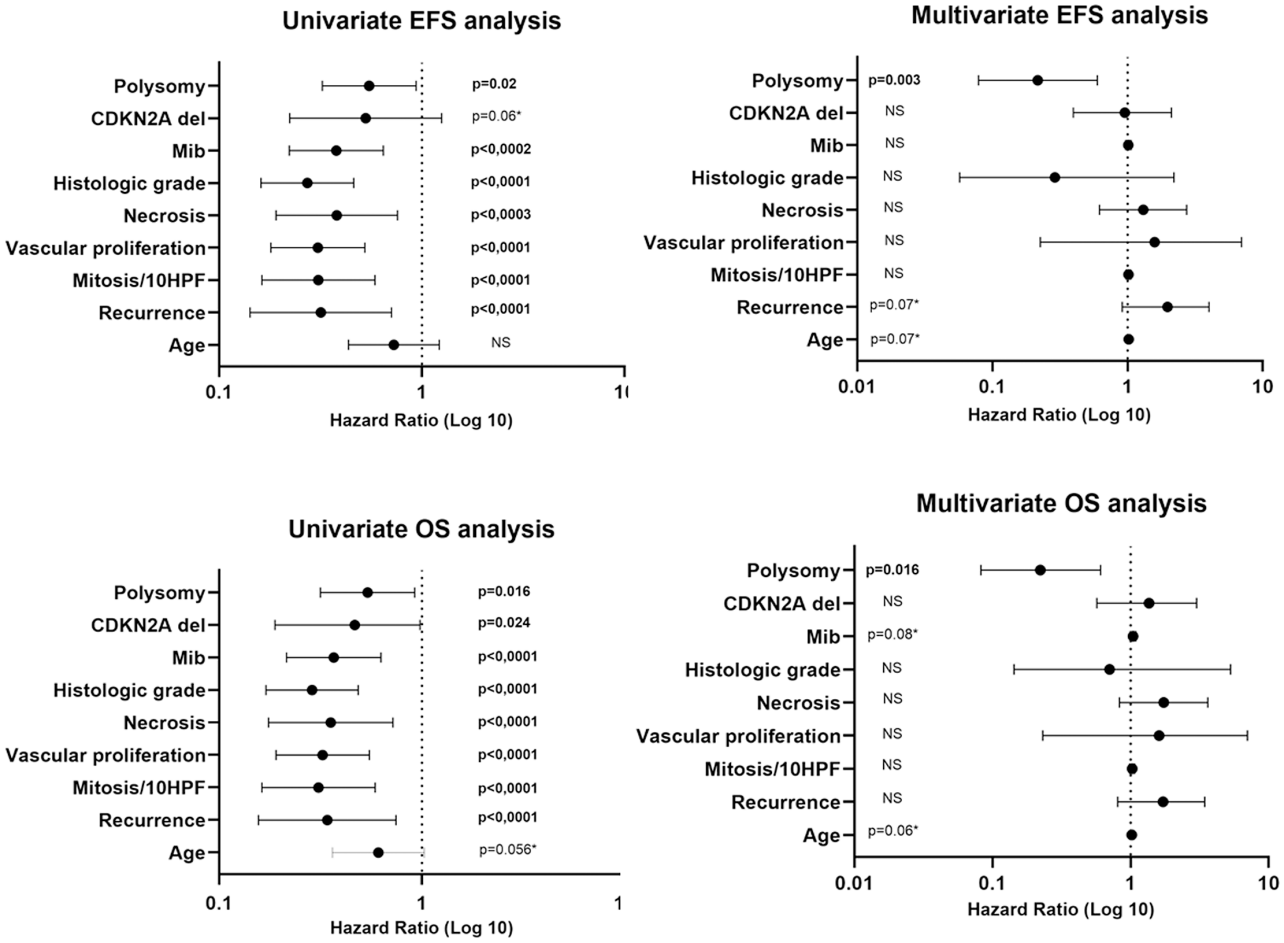
Forest plots of Cox proportional hazard regression analysis for EFS and OS in univariate and multivariate analysis of hazard ratios (HR) and 95% confidence intervals (CI) of features in our cohort. In the multivariate model, polysomy is independently associated with significantly shorter delay before recurrence (EFS) and worse clinical outcome (OS).

1p and/or 19q polysomy was correlated to a shorter EFS in our series (138 months versus 60, p = 0,02). EFS curves revealed a 1-year survival likelihood of 96%, 5-year survival likelihood of 66%, 10-year survival likelihood of 52%, 15-year survival likelihood of 38% and 20-year survival likelihood of 38% when no polysomy was present. When 1p and/or 19q polysomy were noted EFS analysis revealed a 1-year survival likelihood of 82%, 5-year survival likelihood of 47%, 10-year survival likelihood of 31%, 15-year survival likelihood of 31% and 20-year survival likelihood of 31% (Figs 3 and 4, p = 0,02).

In the O3 cohort, the presence of a polysomy was also correlated to a shorter PFS when stratified between 1p/19q copolysomy and single 1p or 19q polysomy (p < 0,0001): table 2 and Fig 4. In the 58 patients with polysomy, O2 had a significant longer EFS than O3 (p = 0,002): Table 2 and Figs 3 and 4.

In multivariate analysis, polysomy appeared to be independently associated with a significantly shorter delay before recurrence (p = 0.003): Fig 3.

### Correlation of clinical, histological and molecular data with OS

In our cohort, older age presented a trend to a significant worse overcome (median of 122 months versus 185, p = 0.056): Table 2.

Kaplan-Meier survival curve analysis revealed a 1-year survival likelihood of 100%, 5-year survival likelihood of 95%, 10-year survival likelihood of 74%, 15-year survival likelihood of 56% and 20-year survival likelihood of 49% for the O2 cohort. We noted a 1-year survival likelihood of 88%, 5-year survival likelihood of 61%, 10-year survival likelihood of 37%, 15-year survival likelihood of 33% and 20-year survival likelihood of 20% with O3 cohort (p < 0,0001, Fig 2).

There was no correlation between the anatomical site, the type of treatment and the presence of calcification with OS in our series (Table 2).

High mitotic and Mib1 proliferative indices were also correlated to lower OS (55 months versus 184, p < 0,0001 and 70 months versus 184, p < 0,0001 respectively) with vascular proliferation (81 versus 193 months, p < 0,0001) and tumor necrosis (60 versus 184 months, p < 0,0001): Table 2 and Fig 4.

CDKN2A deletion was linked to a worse OS (67 months versus 130, p = 0,024). Patients who had no CDKN2A deletion had a median OS of 95% with 1-year, 81% with 5-years, 57% with 10 years, 43% with 15 years and 35% with 20 years whereas patients who had CDKN2A deletion (homozygous or hemizygous) had a median OS of 89% with 1-year, 59% with 5-years and 32% with 10 years (Fig 2, p = 0,02): Table 2 and Figs 2 and 4.

1p and/or 19q polysomy were correlated to a shorter OS (113 versus 175 months, p = 0,016) as compared to non-polysomy. OS curves revealed a 1-year survival likelihood of 99%, 5-year survival likelihood of 85%, 10-year survival likelihood of 65%, 15-year survival likelihood of 48% and 20-year survival likelihood of 38% when no 1p and/or19q polysomy were observed. When polysomy was observed OS analysis revealed a 1-year survival likelihood of 89%, 5-years survival likelihood of 69%, 10-years survival likelihood of 43%, 15 years survival likelihood of 37% and 20 years survival likelihood of 29% (p = 0,016): Table 2, Figs 3 and 4. In the O3 cohort, the presence of a polysomy was also correlated to a shorter OS when stratified between 1p/19q copolysomy and single 1p or 19q polysomy (p < 0,03): Table 2 and Fig 4. In the cohort with polysomy, O2 had a significant longer OS than O3 (p = 0,007): Table 2 and Figs 3 and 4.

In multivariate analysis, polysomy appeared to be independently associated with significantly worse clinical outcome (p = 0.016): Fig 4. Age showed a trend to an independent prognostic factor as well (p = 0.06): Fig 4.

### Concordance between manual and automated analysis of 1p and or 19q polysomy

140 analysis (51 polysomic and 89 non polysomic cases) was fully concordant between manual and automated analysis for 1p signals: concordance of 96% and kappa = 0.92 which correspond to a very high concordance (Table 3).

For 19q signals, 142 analysis (46 polysomic and 96 non polysomic cases) was fully concordant between manual and automated analysis with a concordance of 98% and a kappa = 0.95.

**Table 3:** Concordance between manual and automated analysis in the determination of 1p and 19q polysomy status.

	1p polysomy	19q polysomy
	Manual/ Automated	Manual/ Automated
**Negative cases**	94/ 89	96/ 99
**Positive cases**	51/ 56	49/ 46
**Concordance**	96%	98%
**Coefficient** κ	0,92	0,95

### G/R combinations analysis in polysomic cases

**Fig 5:**
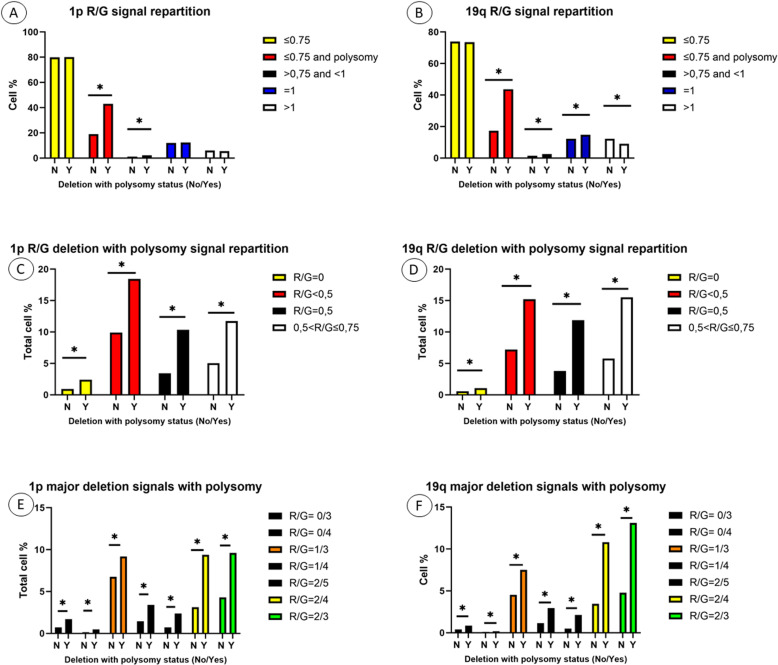
Repartition of R/G signals with 1p/1q and 19q/19p probes obtained by automated FISH analysis. A,B: global R/G signal repartition with signals ≤ 0,75 corresponding to deletion status, between 0,75 and 1 to instability status, = 1 to normal status and > 1 to polysomy without deletion status. C,D: R/G signal repartition within the deletion + polysomy cohort revealed 4 distinct subgroups of signals corresponding to homozygosity for the chromosome (R/G = 0), hemizygosity (R/G < 0,5), polyploidy (R/G = 0,5 with an exception for the 1/2 signature) and instability (R/G > 0,5 and ≤ 0,75). E,F: significant R/G signatures in deletion + polysomic cohort identified 7 major signatures for both Chromosome 1 and 19 probes with a major predominance of R/G = 1/3, 2/4 and 2/3 signatures.

The analysis of all R/G signals distribution in our series revealed some differences between the polysomic and the non-polysomic cohort for both Chr1 and Chr19 signals as summarized in Figs 5A and 5B.

As expected, the distribution of R/G signals for 1p/1q and 19q/19p was quite similar. The majority of all the signals detected (mean = 80% for 1p and mean = 74% for 19q) were deletion signals (R/G < 0.75) as expected according to the WHO molecular definition of OGs for the 1p/19q codeletion. Among these signals, those which corresponded to polysomy, i.e., with a G > 3, represented an average of 19% of total cells for 1p and 17% for 19q in the non-polysomic population while this percentage rises to 43% of the total cells for both 1p and 19q in the polysomic cohort: p < 0.001 (Figs 5A and 5B). We also observed a significant difference in distribution for both 1p and 19q for the R/G signals located between 0.75 and 1 and which correspond to chromosomal instability: mean of 2% versus 1% of the total signals for the polysomic cohort and the non-polysomic one respectively (p < 0.001 for both 1p and 19q). The signals R/G = 1 (corresponding to a normal chromosomal status) and the signals R/G > 1 (corresponding to polysomy without deletion) appeared differently distributed between non polysomic and polysomic cohort only for Chr19 (mean = 12% versus 15%, p = 0.003 and mean = 12% versus 9%, p = 0.015 respectively). This may reflect a greater chromosomal instability affecting more the small Chr19 than the much larger Chr1 (59 million nucleotide base pairs versus 249 million, namely 2% of total DNA versus 8%) in this type of glioma.

In our series, some R/G signals were significantly more present in the polysomic cohort than those without polysomy. These particular signals of polysomy could be categorized into 4 distinct subgroups of signals according to their ratio value:

- Category 1: R/G signals = 0 with a majority of 0/3 and 0/4 signature (which correspond to homozygosity status).
- Category 2: R/G signals < 0.5 with a majority of 1/3, 1/4 and 2/5 (which correspond to hemizygote status).
- Category 3: R/G signals = 0.5 with a majority of 2/4 and 3/6 (which correspond to polyploidy status excepting 1/2).
- Category 4: R/G signals > 0.5 and < 0.75 with a majority of 2/3 and 3/5 (which correspond to an instability status).

For the Chr1, category 1 represented an average of 0.9% of the total signals versus 2.4% (p < 0.0001) between the non-polysomic and polysomic cohorts respectively (Fig 5C). We also noted a significant difference of average in category 2 (10% of the total signal versus 18%, p < 0.0001), in category 3 (3% of the total signal versus 10%, p < 0.0001) and in category 4 (5% of the total signal versus 12%, p < 0.0001): Fig 5C. A similar distribution was noted for Chr 19 with an average of 0.55% of the total signal versus 1.08% (p < 0.0001) for category 1, 7% of the total signal versus 15% (p < 0.0001) for category 2, 3.8% of the total signal versus 11.8% (p < 0.0001) for category 3 and 5.75% of the total signal versus 15, 5% (p < 0.0001) for category 4 (Fig 5D).

More detailed analysis of polysomic signals revealed the presence of seven (7) predominant R/G signals representing almost all of the polysomic signals observed in our study and significantly linked to the polysomic status (Figs 5E and 5F). These signals were distributed as follows: R/G = 0/3 and 0/4 (for category 1), 1/3, 1/4 and 2/5 (for category 2), 2/4 (for category 3) and 2/3 (for category 4). Three (3) of these signals appeared largely predominant representing alone more than 80% of the polysomic signals observed in our series. These are signal 1/3 (which represents 9% of the total signals for 1p and 7.5% for 19q), signal 2/4 (which represents 9% of the total signals for 1p and 11% for 19q) and signal 2/3 (which represents 9% of the total signals for 1p and 13% for 19q): Figs 5E and 5F.

## Discussion

All patients included in our series fulfill the morphological and molecular criteria defining an OG according to the latest WHO recommendations (oligodendroglial morphology, IDH mutation and 1p/19q codeletion) [1].

Similar to data reported by the Central Brain Tumor Registry of the United States (CBTRUS), the majority of our gliomas were located in frontal lobe (77% in our series versus 61% in the CBTRUS) and then in descending order in the temporal lobe (11% versus 13%), the parietal lobe (8% versus 10%) and the occipital lobe (1% versus 1%) [25]. Frontal lobe localization was not correlated to a longer EFS and OS as described in some studies in the literature [26]. As expected, our OGs were present preferentially in adults with a younger age for O2 (median age at diagnosis of 47 years versus 43 years in the CBTRUS dataset) than O3 (median age at diagnosis of 50 years for both our series and CBTRUS dataset) [27]. We also observed a male predominance with an M:F ratio of 1.4:1 versus 1.2:1 as reported in the CBTRUS [27]. For clinical parameters, the only significant difference between O2 and O3 cohorts was noted for postoperative treatment with a higher percentage of untreated patients (patients under monitoring but no planned radiotherapy and/or chemotherapy) in the O2 cohort (46% versus 16%; p = 0,0004). In our series, clinical parameters did not correlate with prognosis, similar to the literature [6]. On the other hand, histological grade was strongly correlated to EFS and OS with a shorter survival delays in O3 cohort as described in literature [28]. According to the WHO CNS5 2021 criteria, high mitotic index, high Mib1 proliferative index, vascular proliferation and tumor necrosis were strongly linked to O3 as observed in our study. We also noted that these histological parameters strongly correlated with a shorter EFS and OS, as reported in the literature [6,29–31]. Intratumoral calcifications were present in 46% of our cases with a significant predominance in O3 cohort but without any correlation with an increased EFS or OS [6].We found a CDKN2A deletion (homozygous + hemizygous deletion) in 13% of our cohort with 7% of homozygous deletion and 9% of hemizygous deletion similar to recent studies [32–34]. Some hemizygous cases were observed in the O2 cohort but majority of them as all homozygous cases were distributed in the O3 cohort as described in the literature [7,32–36]. This CDKN2A/B deletion percentage was similar to recent studies which reported it around 10% in OGs [7,8,32–34] and appeared very low compared to ancient studies and particularly the first ones in which the presence of this deletion was described being more frequent in OGs (between 36 and 55%) [35–38]. This significant variation between studies can be explained by the use of different molecular techniques: FISH [37,38], microsatellite analysis [35], single nucleotide polymorphism (SNP) array hybridizations [7,36], multiplex ligation-dependent probe amplification (MLPA) [33], comparative genomic hybridization (CGH) [7,8]or DNA methylation profiling [34]. For studies using specifically the FISH technique, our low percentage can be explained by our stringent cut off value defining the presence of a deletion (set at 40% instead of 20% [37] and 30% [38]). Our data confirm a strong correlation of the CDKN2A deletion with a shorter OS by univariate analysis as described in literature [7,36–38] but not by multivariate analysis as reported by these studies. This discordance may be explained by our high cut off and our reduced number of homozygote cases. It highlights the need for a better definition of cut offs for the determination of CDKN2A deletion status by FISH as proposed by some authors in IDH mutant astrocytic tumors [39]. Moreover our study demonstrated also the negative prognostic value of the hemizygous deletion in OGs as known for the homozygous deletion [7,36] and as recently debated in the literature for IDH mutated gliomas [32–34]. Further analysis are need to determine the advantages of FISH technique compared to other molecular techniques for detecting these deletions and to establish a consensus on the exact CDKN2A status based on these different method available. Larger data are also need to determine if CDKN2A hemizygous deletion apparition might not be the first indicator of a genomic instability which evolves towards homozygosity as it has been suggested in the progression of IDH mutated astrocytomas [24].

OGs as many human solid malignant tumors are all characterized by chromosomal instability (CIN) which is thought to be an early event during tumorigenesis and tumor initiation. The segmental aneuploidy represented by 1p/19q codeletion is invariably accompanied by heterozygous mutations of IDH1 or IDH2 genes as well as mutations in telomerase reverse transcriptase (TERT) promoter [40]. In a few proportion of OGs, in addition to the segmental aneuploidy (unbalanced number of portions of chromosomes) represented by 1p/19q codeletion, a supplementary whole chromosome aneuploidy (unbalanced number of whole chromosomes) is sometimes also observed in the form of more than 3 copies for 1 q and/or 19p in tumor cells (R/G signals with G > 3). Whole-chromosome aneuploidy is usually caused by a non-disjunction of a pair of homologous chromosomes to separate during meiosis or to a translocation (rearrangement between non homologous chromosomes) whereas segmental aneuploidies refer to unbalanced regions of chromosomes, e.g., caused by deletions, amplifications or translocations. The suffix polysomy is a term frequently used in molecular analysis and particularly in FISH. It is a synonymous of a whole-chromosome aneuploidy and is defined as at least one or more chromosome than normal (that is 3 or more copies of the chromosome rather than the expected two copies). In FISH, polysomy includes cases with underneath deletion or not and by excess and wrongly cases with polyploidy which refers to abnormal number of the complete set of the 23 chromosomes. These two terms of -ploidy and -somy tend more and more to be encompassed in the recent literature by the generic term of CIN [41]. CIN is itself include in a larger definition called genomic instability. The latter englobes CIN but also refers to other forms of presently known genomic instabilities, such as microsatellite instability (MIN) or CpG island methylator phenotype (CIMP). For the sake of clarity, most 1p/19q studies of literature in OGs have preferred to keep the term of “1p/19q-codeletion” for the segmental aneuploidy and to name “polysomy of Chr1 and/or Chr19” this supplementary and occasional whole chromosome aneuploidy (with or without polyploidy). These two anomalies participate independently of each other in the chromosomal instability which is increasingly studied in neoplastic processes nowadays.

Since early 2000, a few studies in the literature have described the presence of a polysomy of chromosomes 1 and 19 in a minority of oligodendroglial tumors [19,42]. Later this polysomy has been associated with concomitant 1p/19 codeletion and to OGs with the evolution of the WHO classification [14–17,20,37,43–45] and than later to 1p/19 codeletion + IDH mutation in OGs [18,46,47]. This association has been called “relative 1p/19q deletion” (by opposition to the cases presenting 1p/19q codeletion only) or “1p/19 deletion with polysomy”. Despite different definitions of this polysomy in these studies published in the literature, the most cited definition and which seems to be the most accepted is that of Snuderl and al [14]. As reported in our previous studies [21,23,38], our laboratory has long implemented automatic analysis of 1p/19q status using FISH technique, with a large collection of archived virtual images since two decades. This gives us the opportunity to get access to a large monocentric series of well molecularly defined OGs. For all our cases, retrospective determination of 1p and/or 19q polysomy status according to the definition of Snuderl et al [14] was easy for both automated and manual analysis. Unsurprisingly it showed a high agreement between these two type of analysis (κ>0.9 for both 1p and 19q) such as observed for the determination of the 1p/19q codeletion status [21]. The rare discordant cases have been false negative cases by manual analysis, which were reclassified positive by automatic analysis on a much larger number of cells allowing the expected cut off of 30% to be reached. The polysomy signals obtained with the commercial probes used in our study were easily distinguishable and accessible to both manual and automatic analysis. Under these conditions, no special adaptation was necessary between these two distinct analysis for any of our cases. Our data confirmed the advantages provided by automatic analysis in time saving (2–5 times less) and number of cells analyzed (up to 10 times more for a constant number of analyzed images). This highlights the exhaustive character of automated analysis in taking into account all the cells detected on the analyzed images. This also explains the concordance difference, even minimal (4% and 2% respectively), between the two analysis for 1p and 19q in our study. This concordance difference between manual and automated analysis appears similar to that observed for 1p and 19q deletion signal analysis in our previous study [21].

Many techniques (FISH, chromogenic in situ hybridisation [CISH], PCR, real-time PCR, multiplex ligation-dependent probe amplification [MLPA], single nucleotide polymorphism [SNP] array, comparative genomic hybridisation [CGH], array CGH, next-generation sequencing [NGS], mass spectrometry and NanoString) allow good sensitivity (few false negatives) for routine detection of 1p/19q codeletions in glioma [13]; but FISH appears the most suitable for detecting this polysomy in the literature, probably because it offers the possibility to analyze the chromosomal status at each cell level. Using 2 separate sets of probes to explore the 2 distinct chromosomes (here Chr1 and Chr19) it also allow a reasonable extrapolation between true polyploidy and polysomy when encountered. Indeed, if both chromosomes 1 and 19 had identical signal pattern greater than 2n, we can consider having a ploidy change; for example, 2R4G for both 1 and 19 is considered tetraploid, 3R6G for both 1 and 19 is considered hexaploid and 2e3R4e6G for 1 and 19 is considered polyploidy (2R5G, 2R6G, 3R4G, 3R5G, 4R4G, 4R5G, 4R/6G). If, however, chromosome 1 and 19 had a distinct signal pattern with signal pattern of 1R2G for one and greater than 2n for the other chromosome, then these cases are to be considered as polysomic [43].

Unlike the majority of FISH studies on 1p/19q polysomy in OGs excluding the single 1p or 19q polysomy cases [14,16–18,20] we decided to keep these cases in our study. This follows other studies [15,44] even if they represent always a minority as in our series (14/58 = 24%) without a great comparison bias risk with the other studies. Despite this choice, we observed the presence of polysomy in 40% of our cohort, similar to the majority of previous studies published in literature in which percentage oscillates between 16% and 59% [14–18,20,37,43,44]. Polysomy were present in both O2 and O3 cohort with a predominance in the O3 population (33% versus 67%) similar to the literature data where the polysomy varies between 10% and 33% for O2 [15–18,20,37,43,44] and between 18% and 73% for O3 [14–18,20,37,43,44].

Our study confirmed the previous and often fragmentary data published on 1p/19q deletion with polysomy: polysomy was strongly correlated with O3 [15,37,43] and for this reason also correlated to histological high grade criteria as mitotic and Mib1 proliferative indices and vascular proliferation. Polysomy was also significantly more common in recurrent OGs as previous studies mentioned [14,37,43]. This polysomy appeared correlated to shorter PFS [14,16,18,20] and OS [15–17,20] and this in univariate and multivariate analysis [16,20].

Within our polysomic cohort, the distribution of R/G signals appeared irregular with a high intratumor variability in the number of copies. Some signatures corresponded to a polyploidy particularly the 2R4G tetraploidy signal (which appeared one of the most encountered signals) whereas other signatures corresponded to polysomy (with the most frequent signature corresponding to the 2R3G). Like other studies, all our cases presented a systematic mixture of polysomy and polyploidy signal with very few pure polyploid cases as reported in literature [14,15,43]. This suggest that polysomy is not secondary to tumor polyploidy (or hyperploidy) but rather a tumor-associated alteration and an evidence of an increasing genomic instability which correlates with tumor progression in OGs [48] and as observed in IDH-mutant astrocytomas [49]. This genomic instability remaining the same as the initial glioma in the majority of recurrent cases as observed in our series (8/13 = 61%) but getting more complex in a significant proportion of the others (4/13 = 31%). Only a minority seemed to evolve favorably towards a more quiescent chromosomal instability profile (1/13 = 8%).

Moreover our study had shown that the large majority of polysomic signals encountered can be easily reduced to seven (7) predominant R/G signals. This small number of signal allowing to implement them easily in the preexisting algorithm of automated analysis software [21].

Our results, although encouraging, are limited to cases from a single institution. They need to be validated by other institutions that do not necessarily use the same molecular technique as ours or the same FISH probes or even the same FISH signal analysis interpretation. It would be particularly valuable for future studies of polysomy in oligodendrogliomas if experts in the field could agree on the threshold for reporting 1p and 19q polysomy status for each molecular technique used in CNV analysis [13].

## Conclusion

This study confirms the prognostic value of chromosome 1 and/or 19 polysomy in OGs in a large monocentric and retrospective series. This polysomy is strongly correlated to high grade OG status and with decreased EFS and OS by univariate and multivariate analysis. Automated determination of 1p and/or 19q polysomic status is easy to perform and shows a very strong concordance with manual analysis. Moreover our study shows that the large majority of polysomic signals can be categorized by a small number of R/G signals easily implementable to FISH automated analysis software and allowing future multicentric comparisons with better turnaround times and decreased technical costs. These data emphasized the necessity to report the polysomy status, whether copolysomy or single polysomy, of chromosomes 1 and/or 19 for all primary and recurrent gliomas submitted in routine practice for chromosome 1p/19q deletion analysis by FISH. They also raise the possibility that the analysis of this additional chromosomal anomaly could be introduced into the quality assurance programs of FISH technique for 1p and 19q deletion in gliomas, which may encourage its wider utilization.

## Supporting information

S1 Table Global clinical, histological and molecular data of the study. (XLSX)
